# Increased expression of hypoxia inducible factor-1 alpha and vascular endothelial growth factor is associated with diabetic gastroparesis

**DOI:** 10.1186/s12876-020-01368-y

**Published:** 2020-07-10

**Authors:** Xueping Wu, Zhifang Yang, Zhihong Li, Ling Yang, Xinyan Wang, Congrong Wang, Jun Gu

**Affiliations:** 1 Department of Anatomy, Histology and Embryology, Shanghai University of Medicine & Health Sciences, 279 Zhouzhu Road, Pudong New District, Shanghai, China; 2 Department of Physiology, Shanghai University of Medicine & Health Sciences, 279 Zhouzhu Road, Pudong New District, Shanghai, China

**Keywords:** Diabetic gastroparesis, Streptozotocin, HIF-1α, VEGF

## Abstract

**Background:**

Gastroparesis is a recognized complication of diabetes but its pathogenic mechanism incompletely understood. Our aim was to determine whether HIF-1α and VEGF are secreted from gastric tissue is a fundamental factor that drives diabetic gastroparesis.

**Methods:**

Diabetes was induced in Sprague-Dawley by a single intraperitoneal injection of 65 mg/kg streptozotocin. After 4 and 12 weeks, rats were euthanized for assaying body weight, blood glucose, gastric acid secretion and gastric emptying. Morphologic changes in gastric mucosa were observed by the light microscope. Expression of HIF-1α and VEGF were assessed using immunohistochemistry, RT-PCR and Western blot analyses.

**Results:**

Compared with control group, blood glucose were significantly increased and body weight were markedly decreased in streptozotocin-induced diabetes. Gastric emptying was significantly decreased in diabetic rats compared to the control group at different times. The number of parietal cells was obviously decreased, and vacuolated degeneration in diabetic rats. Gastric acid secretion in diabetic group was significantly decreased, and expression of HIF-1α and VEGF were significantly increased in the diabetic group.

**Conclusion:**

These results indicated that overexpression of HIF-1α and VEGF in the gastric mucosa and played a pivotal role in the progression of diabetic gastroparesis.

## Background

Diabetic gastroparesis (DGP) is a chronic and potential complication of diabetes [1]. However, Type 1 diabetes mellitus (T1DM) patient had more serious gastric emptying delays [2] lead to unsatisfactory glycemic control [3] and may be the first sign that the patient is developing gastroparesis.

The pathophysiology of DGP is a complex and multifactorial and remains to be determined, several studies suggested that DGP is associated with hyperglycemia, changes in gastrointestinal hormones, and microvascular lesions [4]. In the stomach, parietal cells are an established source of secreted acid and appear to be key regulators of gastric gland homeostasis. More importantly, parietal cells are not only play an important act in maintaining the normal structure and function of gastric mucosa, but also play a major role in regulating gastrointestinal motility [5]. Chronic DM leads to marked changes in gastrointestinal function, decreased gastric secretion and impaired glycemic control in diabetes [6]. However, whether there is any correlation between the change of parietal cells function and DGP requires further investigations.

HIF-1α and VEGF are closely related to diabetic complications [7]. Long-standing hyperglycemia promotes synthesis and secretion of VEGF, which was transcriptional regulated by HIF-1α, as the major growth factor mediating vascular leakage and neovascularization [8]. Forasmuch as the gastric mucosa are also a major source of VEGF and HIF-1α [9]. Therefore, glucose-dependent transcriptional dysregulation of VEGF is a likely mechanism to promote the occurrence of DGP. Previous studies showed that transgenic mice overexpressing VEGF induced hyperpermeable vessels [10]. Despite its importance in blood vessel formation, the exact molecular mechanism responsible for VEGF in diabetic tissue remains unknown. Very few studies have been performed to show whether morphological changes in the parietal cells, and the relationship between the changes of HIF-1α and VEGF expression in stomach and the pathological course of DGP in STZ-induced diabetic rats. Thus, in present study, we explored the morphological and changes of the expression of HIF-1α and VEGF in stomach of diabetic rats, and to provide a morphological basis for in-depth study of the role and significance of HIF-1α and VEGF in the pathogenesis of DGP.

## Methods

### Experimental animals

All experiments were performed on male Sprague-Dawley rats (8 weeks, *n* = 60, 180–220 g, obtained from SLAC National Rodent Laboratory Animal Resources (Shanghai, China). The rats were housed in individual cages with ad libitum access to food and water and maintained under a controlled environment (24 °C, humidity 60 ± 10%, 12/12 h light/dark cycle).

### Induction of diabetes

All rats were anesthetized with intraperitoneal injections of pentobarbital sodium (50 mg/kg body weight). Type 1 diabetes was induced by a single intraperitoneal (i.p.) injection of STZ (Sigma-Aldrich, St Louis, MO, USA) at 65 mg/kg (*n* = 48), prepared in 0.1 M citrate buffer (CB; pH 4.5). Rats in the control group were injected with equal volume of 100 mM citrate buffers (pH 4.5) (*n* = 12), after overnight fasting. Blood glucose level was determined at the point of the experiment from the tail vein using a blood glucometer (Life Check, Gunze, Kyoto, Japan). Animals with a level of > 16.7 mmol/L were considered as hyperglycemic and were placed in the experiment type 1 DM group (Among them, 6 rats died after STZ injection, survival rats were randomly divided into 4-week and 12-week groups with 21 rats in each group). At the end of week 4 or 12 W, the rats were euthanized with intraperitoneal injections of pentobarbital sodium (100 mg/kg), serum and gastric tissues were collected from the animals for biochemical assays.

### Measurement of blood glucose and body weight

The weight was measured at the beginning of the experiment, and at the end of the 4 and 12 weeks and anesthetized with pentobarbital intraperitoneally. The abdominal cavity was opened and 5 ml blood was quickly drawn from inferior vena cava for blood glucose detection.

### Gastric acid measurements

Gastric acid secretion was determined as described previously [11]. The rats were fasted for 24 h. Rats in the three groups were anaesthetized and executed. Gastric cardia and pylorus were clamped, and the stomach was removed, immersed in 2 ml of oxygen-saturated normal saline and opened along the lesser curvature. Collect the solution containing gastric contents, centrifuge 500 g for 5 min, use Topfer’s as an indicator, titrate 0.1 mL supernatant with 0.01 mmol/L NaOH, total gastric acid secretion is expressed as total acid output [μEq/(L·kg)].

### Detection of pigment residual rate in stomach

The rate of gastric emptying was determined by a modification as described previously [12]. Methylene blue was added to methylcellulose solution with a concentration of 1.5% after 24 h of fasting, and the final concentration was 1 mg/mL. Methylene blue solution 0.4 ml was infused a stainless steel feeding tube. Rats were returned to their cages without food or water and euthanized 30 min later. The abdominal cavity was opened, the stomach was clamped at the gastric cardia and pylorus, and the stomach removed. It was then open and the gastric contents were rinsed in 4 mL saline solutions. The rinsing solution was collected and centrifuged at 3500 rpm for 15 min. Absorption value (OD) was measured at 620 nm of 721-A spectrophotometer (Bio-Rad, USA). The residual rate of gastric pigments was calculated according to the residual methylene blue. The gastric emptying of rats was expressed by the residual rate of gastric pigments. The residual rate of gastric pigments was equal to the OD value of the determination tube/the OD value of the standard tube × 100%.

### Histology

Samples were processed for histological and immunohistochemical examinations at 4 and 12 W after injection of STZ or citrate buffer. The body of the stomach from control, 4 W and 12 W diabetes rats were romoved and fixed in 4% paraformaldehyde and embedded in paraffin, sectioned at 5 μm, and stained with hematoxylin and eosin (H&E). Histological analyses the mucosal thickness and the changes of parietal cells. Parietal cells were counted in H&E-stained sections taken from every rat used in the study. The number of parietal cells was performed on at least ten sections taken at random from the gastric mucosa of normal and diabetic rats using Image J Software (NIH, Bethesda, USA). The thickness of the gastric mucosa was measured on at least six sections per animal. Sections were viewed and photographed under the microscope with the × 10 (visual field diameter, 2.5 mm).

### Immunohistochemistry

Sections were cut at 5 μm, and mounted on poly-L-lysine-coated slides. Sections were deparaffinized, dehydrated through a graded xylene-alcohol series, and washed in tap water. Endogenous peroxidase activity was inactivated with 0.3% H_2_O_2_ for 30 min. After being washed with phosphate buffered saline (PBS). Slides were incubated with normal goat serum to block non-specific binding of the primary antibody for 45 min at 37 °C in a humidified container. The blocking serum was removed by gentle tapping, and slides were incubated with either rabbit polyclonal anti-HIF-1α (sc-10,790, 1:100, Santa Cruz, CA, USA) or mouse monoclonal anti-VEGF (sc-7269, 1:100, Santa Cruz) for overnight at 4 °C in a humidified container. Sections were incubated with a secondary goat anti-mouse (sc-2005, 1:500, Santa Cruz) or anti-rabbit antibody (sc-2004, 1:500, Santa Cruz) for 60 min and washed three times in PBS. Incubation with DAB solution for 10 min and then sections were weakly counterstained with hematoxylin. The same concentration of normal rabbit immunoglobulin G was used as a negative control. The slides were cover-slipped with neutral gum mounting and examined under an optical microscope (OlympusX71, Tokyo, Japan). Positive staining (dark brown) was quantified by two investigators in a blinded manner under high-power magnification (× 400) using the image analysis software Image-Pro Plus 7.0. The data are expressed as the average intensity of the threshold area. Ten horizons (400) were randomly selected in each slice and photomicrographed. Appearance of brown granules in the target cell was considered as a positive reaction.

### RT- PCR

Total RNA of gastric tissues was extracted using TRIzol reagent (Invitrogen, CA, USA). RNA was reverse transcribed using a reverse transcription kit (TaKaRa, Otsu, Shiga) according to the manufacturer’s instructions. The primers (Sangon Biotech, Shanghai, China) were (5′-3′): HIF-1α-fw: AAGTCTAGGGATGCAGCA, HIF-1α-rev: CAAGATCACCAGCATCTAG, VEGF-fw:CGAGACGCAGCGACAAGGCA,VEGF-rev: ACCTCTCCAAACCGTTGGCACG, β-actin-fw: GTGTGACGTTGACAT, β-actin-rev: ACATCTGCTGGAAGGTG. The amplification process was performed on a realtime PCR instrument (RotorGene 3000, Australia). The PCR products of each group were analyzed by 1% agarose gel electrophoresis and visualized with ethidium bromide staining, then the image analysis and data processing were carried out by gel imaging analysis system (Furi Science & Technology, Shanghai). The mRNA expression levels were normalised to β-actin mRNA levels.

### Western blot

The gastric tissues of each rat in different group were treated with 100 μl RIPA lysis buffers (Pierce, Rockford, IL) and lysis on ice for 30 min. Upon centrifugation at 14,000 g for 10 min at 4 °C, the supernatant was collected, and the total protein was gauged using a BCA protein assay kit (Beyotime, Shanghai) following the manufacturer’s manual. 10% SDS-PAGE gel electrophoresis for 2 h, transfer the protein to the PVDF membrane (Amersham, England) with a wet electric rotary instrument. Under the room temperature condition, the PVDF membrane was blocked by 5% BSA Tris buffer salt solution (tris-buffered saline Tween-20, tris-buffered). Rabbit polyclonal anti-HIF-1α antibody (1:1000, Santa Cruz), mouse anti-VEGF monoclonal antibody (1:1000, Santa Cruz) was added and incubated overnight under 4 °C. TBST was used to wash the membranes 5 times, 6 min/time, then incubated with HRP-conjuncted goat anti-mouse antibodies or goat anti-rabbit (1:2000, Santa Cruz). Chemiluminescent signals of protein bands were visualized using the enhanced chemiluminescence (Bio-Rad, Hercules, CA). Densitometry analysis was performed using the ImageJ software. β-actin was used as the internal reference.

### Statistical analysis

The datum was analyzed using SPSS (version 21.0; IBM Corporation, NY, USA). Results were given as means ± SE. Statistical analysis was performed by using Student’s unpaired t-test and one-way ANOVA with Scheffe’s post-hoc multiple-comparison analysis. Correlations between the two factors was analyzed using Pearson’s correlation analysis. A value of *p* < 0.05 was considered statistically significant.

## Results

### Changes in body weight, blood glucose, gastric acid secretion and gastric emptying in STZ-induced DM rats

**Fig. 1:**
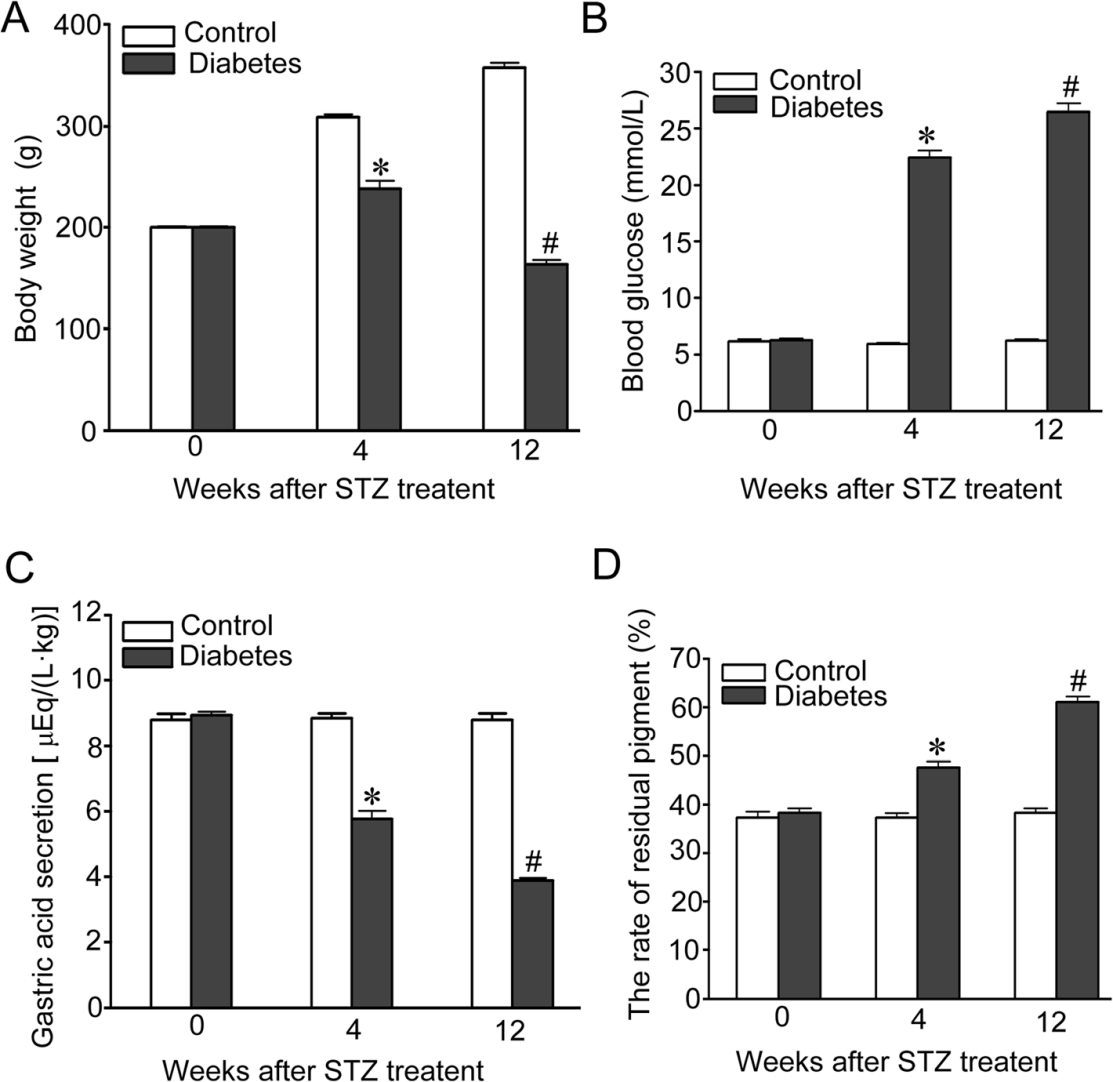
The change of body weight, blood glucose, gastric acid secretion and gastric emptying after induction of diabetes by STZ. Body weight (**a**) and blood glucose level (**b**) were measured in the control and diabetic rats. Gastric acid secretion (**c**) and Gastric emptying (**d**) were assessed in control and diabetic rats. Data are shown as mean ± SEM, *n* = 10 rats per group. *p < 0.05 vs the control group. ^#^*p* < 0.05 vs the 4 W diabetic group

In diabetic rats, markedly decreased in body weight at the end of weeks 4 and 12 was observed compared with the control group (Fig. 1a). The blood glucose level was incessantly elevated (> 16.7 mmol /L) in the DM group, starting from 3 days after STZ administration until the end of the experiment (4 and 12 W) (Fig. 1b) (*p* < 0.05 vs. Normal control group). Figure 1c shows the effect of diabetes on gastric acid secretion, compared with the control group, gastric acid secretion was significantly reduced in diabetic rats and gastric acid remained marked (*p* < 0.05) reduced until the end of the expriment. Figure 1d shows the residue of gastric pigment was increased in the diabetic group (*n* = 12) compared with normal controls and the residue of gastric pigment remained marked (p < 0.05) increased until the end of the expriment.

### Morphology assessment of diabetic gastric tissue

**Fig. 2:**
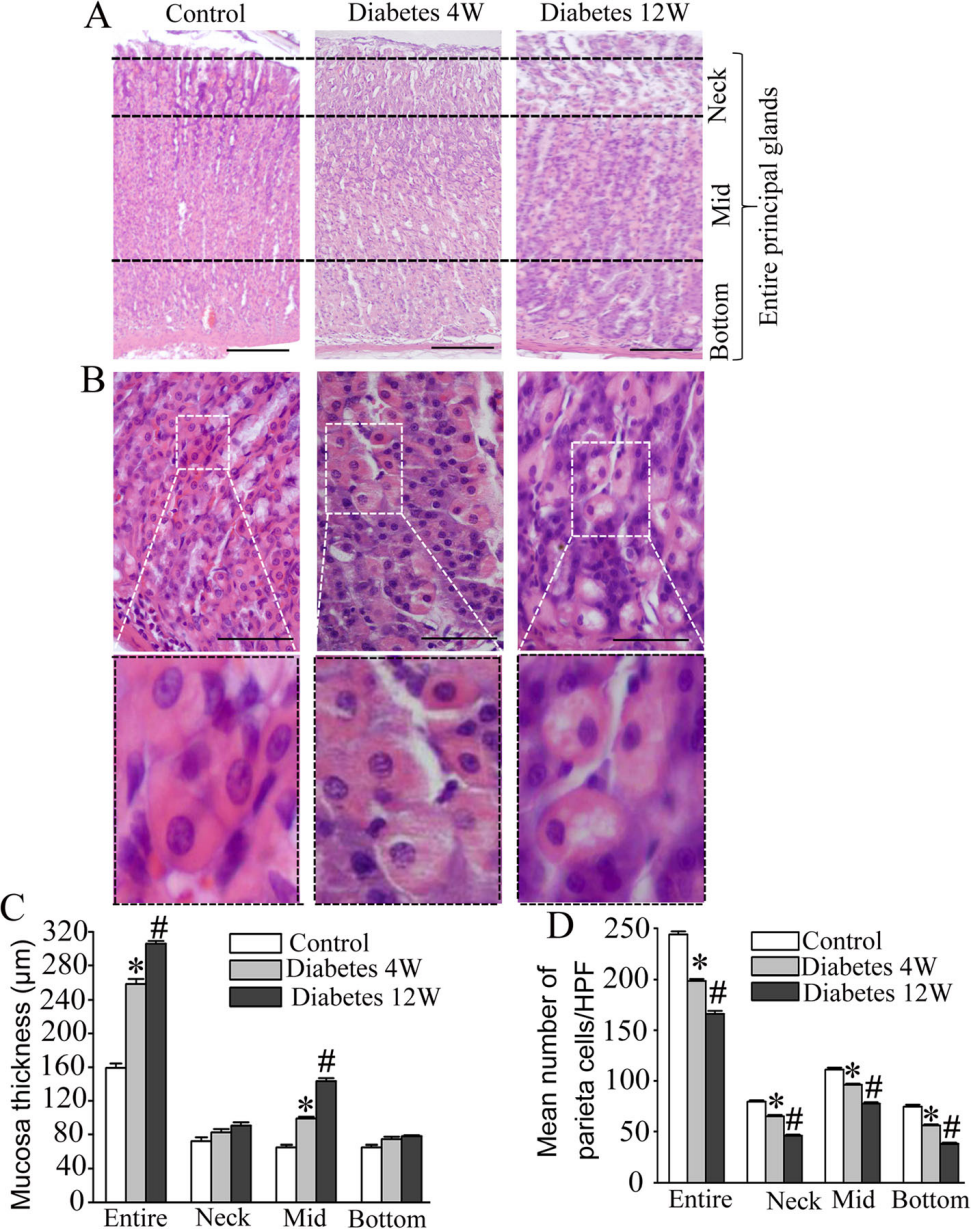
The changes of gastric mucosa morphology after induction of diabetes by STZ. **a**. H&E, × 100. **b**. Mucosa thickness. Data are shown as mean ± SEM, n = 10 rats per group. *p < 0.05 vs the control group. # *p* < 0.05 vs the 4 W diabetic group. **c**. H&E, × 400. Boxed areas depict regions enlarged. Stomach of diabetic rats showing that the parietal cells were irregularly scattered, enlarged and cytoplasmic vacuoles when compared to normal control. **d**. The number of parietal cells was counted in H&E stained sections. Data are shown as mean ± SEM, *n* = 10 rats per group. *p < 0.05 vs the control group. ^#^*p* < 0.05 vs the 4 W diabetic group

The overall morphology and the thickness of gastric mucosa in normal control group appeared normal in H&E-stained sections, the entire mucosa divide into three areas named neck, mid and bottom areas (Fig. 2a). Figure 2c showed that entire mucosal thickness significantly increased in 12 W diabetic rats compared with control group. And the mucosal thickness significantly increased in mid and bottom area of mucosa compared with contorl group. No significant difference was found between 4 W diabetes group and a control group. The size of parietal cells in 12 W diabetic rat was significantly bigger than those of control group. The parietal cells were irregularly scattered, increased in size and vacuolated degeneration as showed in Fig. 2b, significant loss of parietal cells in the principal gastric glands was observed in diabetic rats at 4 and 12 W when compared with control group (*p* < 0.05). And the loss of parietal cells markedly decreased in neck and mid area of glands compared with contorl group (Fig. 2d).

### Immunohistochemical detection of HIF-1α and VEGF expressed in rat gastric mucosa

**Fig. 3:**
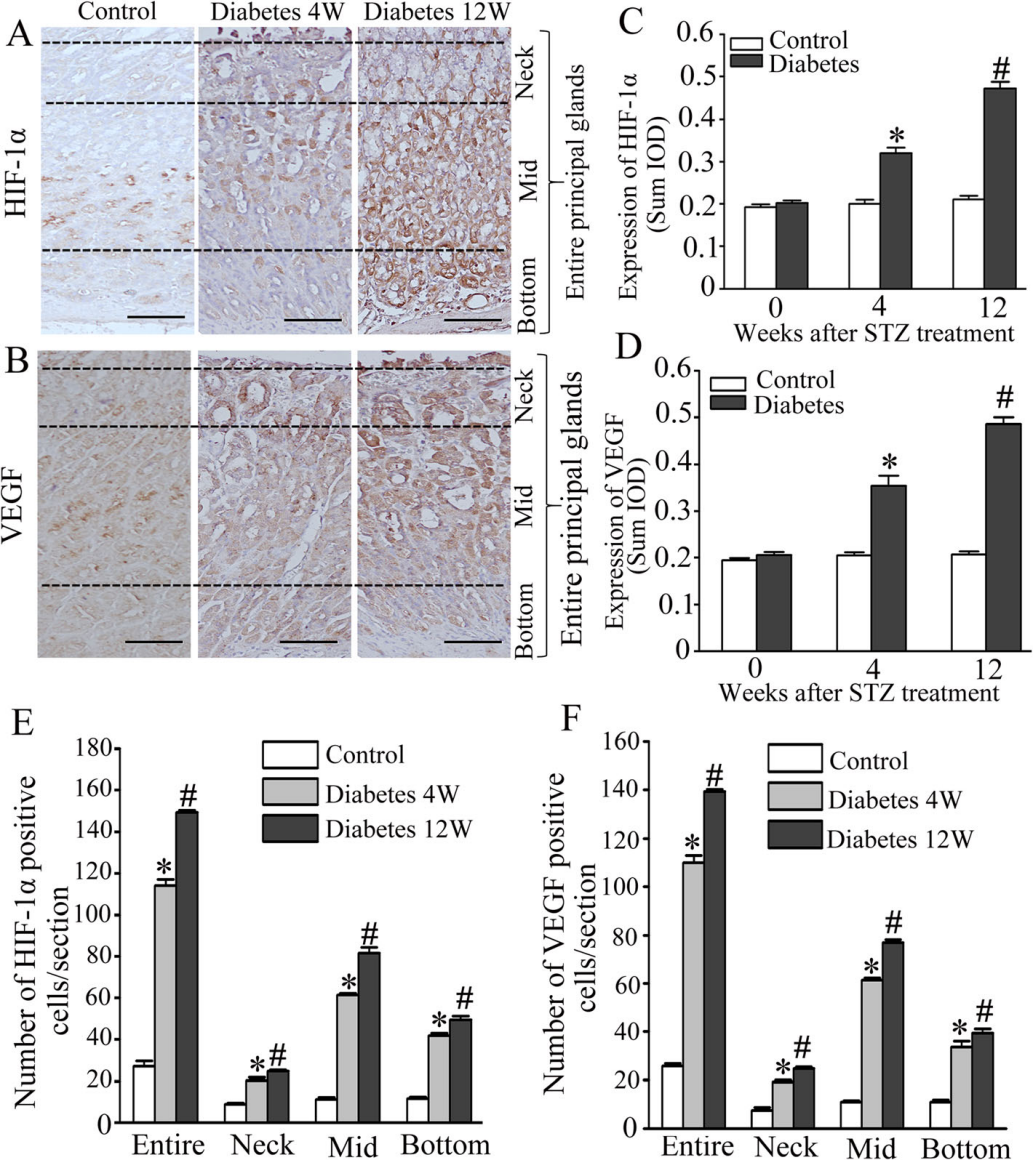
Opposite expression of HIF-α and VEGF in glandular stomach of rats after diabetes induction. (magnification 20 ×). Semiquantitative immunohistomorphological analysis of glandular HIF-α and VEGF expression of diabetic and control rats. The arbitrary lines presented in illustrations (**a**) and (**b**) divide the entire gastric glands into three areas named neck, mid, and bottom areas. The neck area includes very narrow isthmus and neck areas of gastric glands; mid area, the upper area of gastric gland base; bottom area, the deep area of gastric gland base. **a**, **c**, **e** Semiquantitative immunohistomorphological analysis of glandular VEGF expression in diabetic and control groups. **a** HIF-α immunostained longitudinal sections of glandular stomach mucosa (magnification × 20) demonstrate general increase of stain intensity of HIF-α immunoreactive glandular cells (brown color) most prominently in the neck and the bottom of principal glands of diabetic compared with control groups. **c**: SUM IOD of HIF-1α expression level in control, 4 W and 12 W diabetic gastric tissue. **e** Significantly increased stain intensity of HIF-α immunoreactive cells in neck and the bottom area of principal glands of diabetic (black gray columns) compared with control (white columns) groups. **b**, **d**, **f** Semiquantitative immunohistomorphological analysis of glandular VEGF expression in diabetic and control groups. **b** VEGF immunostained longitudinal sections of glandular stomach mucosa (magnification × 20) demonstrate a gradually increase stain intensity (most prominent in the neck and Mid area) of VEGF immunoreactive glandular cells (brown color) in diabetic compared with control groups. **d** SUM IOD of VEGF expression level in control, 4 W and 12 W diabetic gastric tissue. Data are presented as mean ± SD. Data are shown as mean ± SEM, *n* = 10 rats per group. *p < 0.05 vs the control group. ^#^*p* < 0.05 vs the 4 W diabetic group. Bar = 50 μm

HIF-1α and VEGF positive immunoreactivity were detected in the nucleus and cytoplasm. In the sections of STZ-induced diabetic rats, strong HIF-1α and VEGF immunoreactivity were found in the nucleus and cytoplasm of the fundus mucosa (Fig. 3a and b). As we have shown, STZ-induced diabetic rats demonstrated enhanced HIF-1α or VEGF expression level in the fundus mucosa (*p* < 0.05) compared with control group (Fig. 3c and d). Compared with control group, the number of HIF-1α positive cells in the principal gastric glands were significantly increased in the diabetic rats (*p* < 0.05). These changes are most prominent in the mid and bottom areas of the principal gastric glands compared with contorl group (Fig. 3e). Compared with control group, the number of VEGF positive cells in the principal gastric glands were significantly increased in the diabetic rats (*p* < 0.05), which was most prominent in the neck and mid areas of the principal gastric glands compared with contorl group (Fig. 3f). The specificity of staining was confirmed by secondary antibody alone (data not shown).

### RT-PCR and western blot detection of HIF-1α and VEGF expression in rat gastric mucosa

**Fig. 4:**
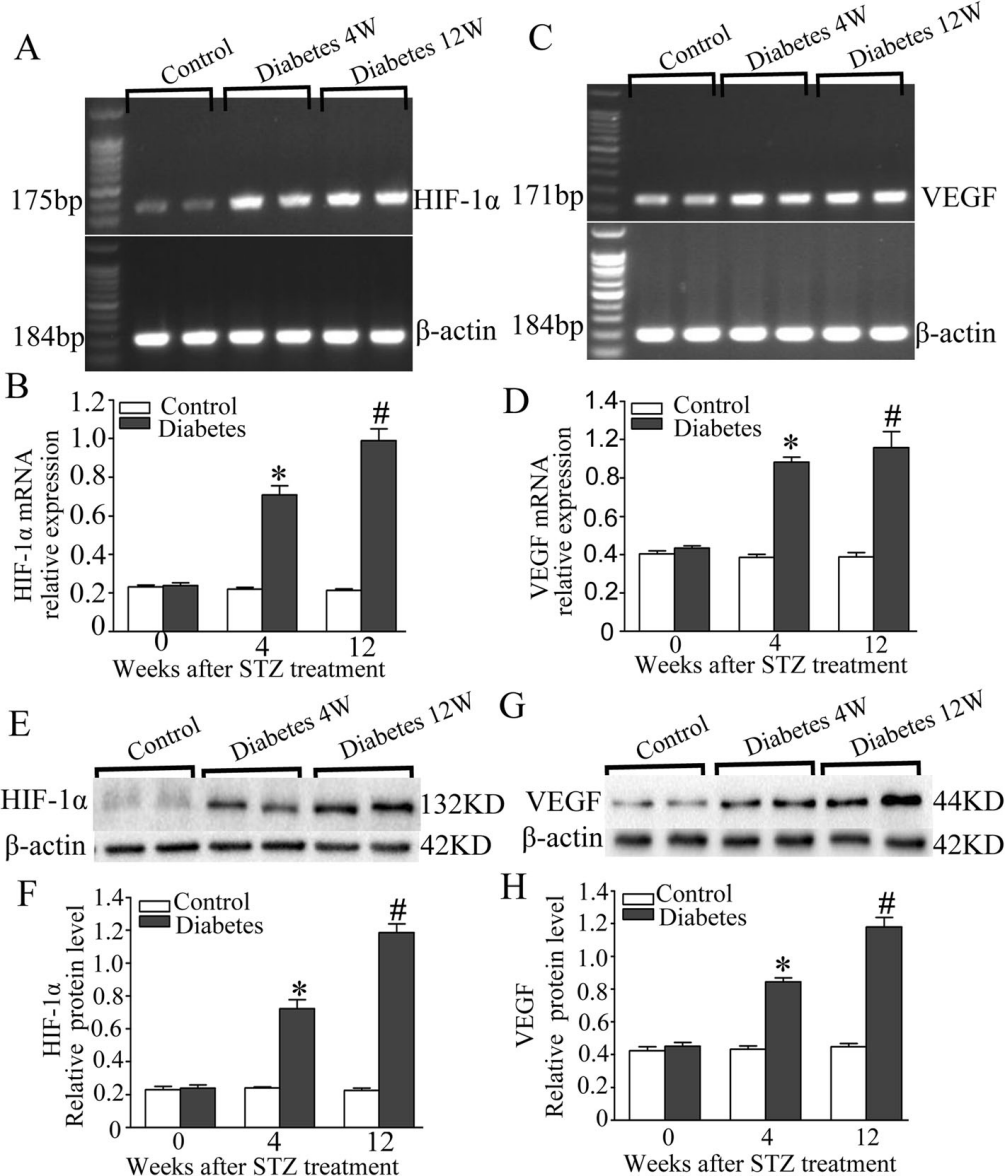
HIF-1α and VEGF expression in rats gastric tissue after induction of diabetes by STZ. **a**. RT-PCR analysis of HIF-1α mRNA expression. **b** The histogram of the gray values of relative HIF-1α mRNA expression. **c** RT-PCR analysis of VEGF mRNA expression. **d** The histogram of the gray values of the relative VEGF mRNA expression. **e** Western blot analysis of HIF-1α protein expression. **f** The histogram of the gray values of relative HIF-1α protein expression. **g** Western blot analysis of VEGF protein expression. **h** The histogram of the gray values of the relative VEGF protein expression. Data are shown as mean ± SEM, n = 10 rats per group. *p < 0.05 vs the control group. # *p* < 0.05 vs the 4 W diabetic group

Analysis of the grey values with image analysis software showed that the expression levels of HIF-1α and VEGF. Results shows that compared with control group, expression of HIF-1α mRNA (Fig. 4a, b) and protein (Fig. 4e, f) and VEGF mRNA (Fig. 4c, d) and protein (Fig. 4g and h) in the diabetic rats were significantly higher than those in control group at 4 and 12 W.(*p* < 0.05).

### Correlation analysis of HIF-1α and VEGF protein expression level

**Fig. 5:**
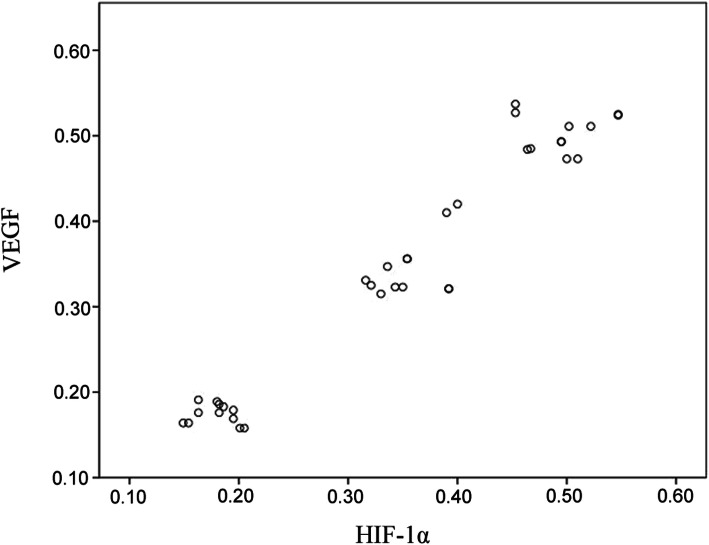
Correlation analysis of HIF-1α and VEGF protein expression. Scatter plot of HIF-1α and VEGF protein expression level were subjected to Pearson’s correlation analysis. Data are shown as mean ± SEM, *n* = 12 rats per group. Significant at the *p* < 0.01 level

In diabetic and control groups, HIF-1α and VEGF protein expression level were subjected to Pearson’s correlation analysis. HIF-1α and VEGF protein expression level were positively correlated with each other (Fig. 5).

## Discussion

Gastroparesis is one of the most common complications of diabetes, accounting for 50% of long-term diabetic patients [5]. The present results clearly showed that gastric emptying was remarkably lower in STZ-induced diabetic rats. Furthermore, we also found that the thickness of gastric mucosa prominently increased in diabetic rats [13]. Delayed gastric emptying is now recognized as part of a broader spectrum of gastric neuromuscular dysfunction that includes impaired gastric accommodation [14]. However, little attention has been paid to the morphology and function of parietal cells in the development of delayed gastric emptying.

The number of parietal cells is significantly decreased and irregularly scattered, enlarged and vacuolated degeneration and swollen in diabetic rats. This is in accordance with previous studies that long-standing diabetic conditions have detrimental influence on the gastrointestinal tract [15]. Parietal cells play critical roles in regulating cellular growth and cell lineage differentiation in gastric mucosa. However, the differentiation and proliferation of the parietal cells in the isthmus of the fundus gland may be inhibited. The inhibition of these functions could play an important role in the pathogenesis of gastric mucosal atrophy and gastric acid secretion in diabetic patients and partly attribute to gastroparesis in DM [3].

Gastric acid secretion reduced and impedes gastric emptying [16]. However, there are no scientifically proven date to show the histological structures and functions of parietal cells in the stomach of STZ-induced diabetic rats are associated with the gastric acid secretion and motility impaired diseases. This study demonstrated that gastric acid secretion was significantly reduced in STZ-induced diabetic rats at different time compared with the control. This result is in consistent with previous studies that in diabetic condition, gastric acid secretion in parietal cells is remarkably decreased in both human and animal studies [17, 18].

Research shows that delayed gastric emptying to be associated with increased production of inflammatory factors [19]. The cytokines produced by mucosal cells, such as HIF-1α,VEGF, interferon (IFN)-γ, tumor necrosis factor (TNF)-α, and IL-10, are contributing to metaplasia in several tissues, including the stomach [8]. Up to the present time, HIF-1α and VEGF were mainly explored in diabetic retinal injury [20], diabetic cardiomyopathy [21], no report has demonstrated the expression of HIF-1α and VEGF in diabetic gastrointestinal mucosa. In the present study, we confirmed that the expression of HIF-1α and VEGF in gastric mucosa was increased significantly in STZ-induced diabetic rats. These data suggest that gastric mucosa may also express the HIF-1α and VEGF and take part in the multifunctional actions in the stomach mucosa. This is a new biological marker of diabetic gastric mucosa in vivo.

It has been accepted that the intensity of HIF-1α expression is closely related to the degree of hypoxia and the condition of tissue ischaemia [8]. The expression of HIF-1α in the gastric mucosa of diabetic rats increased with the prolongation of the course of the disease, suggesting the existence of hypoxia in the gastric mucosa. We hypothesized that hyperglycemia induced hypoxia in local gastric epithelial cells. The expression of HIF-1α was upregulated and the downstream genes were sequentially expressed, resulting in repeated injury, repair and proliferation of the local gastric epithelial cells. It is suggested that hypoxia induced up regulation of HIF-1α may be the initiating factor or key step of delayed gastric emptying. It may play an important role in the occurrence and development of DGP.

The expression pattern of VEGF, whose transcription is regulation and control in an HIF-1α dependent manner. A correlation exists between VEGF and HIF-1α expression, suggesting the justification of our results. Hyperglycemia increased VEGF promoter activity and upregulated VEGF transcript and protein production [22]. It has a high affinity with vascular endothelial growth factor receptor in endothelial cells. It can promote the increase of microvenous and small vein permeability, vascular endothelial cell division, proliferation, calcium aggregation of cytoplasm, and angiogenesis [23]. It plays a key role in the process of various diseases. Diabetes causes severe pathological changes to the microvasculature in many organs and tissues [24]. It plays a key role in the process of complication of DM [25]. At the same time, the high expression of VEGF in gastric mucosal vessels indicates that VEGF can also increase the permeability of gastric mucosal vessels and increase the risk of bleeding in gastric mucosa. These results may be associated with an increase in the thickness of the gastric mucosa.

In addition, the number of glands decreased, the volume of the parietal cells increased and marked vascular degeneration. These alterations are closely related to the increase of gastric mucosal thickness. It is concluded that long-term diabetes induces cellular and functional changes in the glandular stomach especially in the parietal cells. This suggests that the high expression of HIF-1α and VEGF is associated with the aggravation of diabetic gastric mucosal lesions. Therefore, we think that HIF-1α and VEGF can be used as a monitoring index to reflect the severity of the disease, so as to provide useful clues for clinical prevention and diagnosis.

## Conclusion

Our study demonstrates that the HIF-1α and VEGF expression is upregulated in gastric tissue of diabetic rats. The results suggest that HIF-1α and VEGF are involved in the development of gastroparesis in STZ-induced diabetic rats.

## Supplementary information

**Additional file 1.** Entire RT-PCR and Western blot date of HIF-1a and VEGF.

**Additional file 2.** A. RT-PCR analysis of HIF-1α mRNA expression. B. Western blot analysis of HIF-1α protein expression. C. RT-PCR analysis of VEGF mRNA expression. D. Western blot analysis of VEGF protein expression.

## Data Availability

The data can be available from authors upon request.

## Abbreviations

HIF-1α: hypoxia inducible factor-1 alpha
VEGF: vascular endothelial growth factor
DGP: Diabetic gastroparesis
DM: diabetes mellitus
STZ: Streptozotocin
PBS: phosphate buffered saline
