# Supplementary figures and images for: Increased expression of hypoxia inducible factor-1 alpha and vascular endothelial growth factor is associated with diabetic gastroparesis
Source: BMC Gastroenterol. 2020 Jul 10;20:216. doi: 10.1186/s12876-020-01368-y (PMC7350597; doi:10.1186/s12876-020-01368-y)

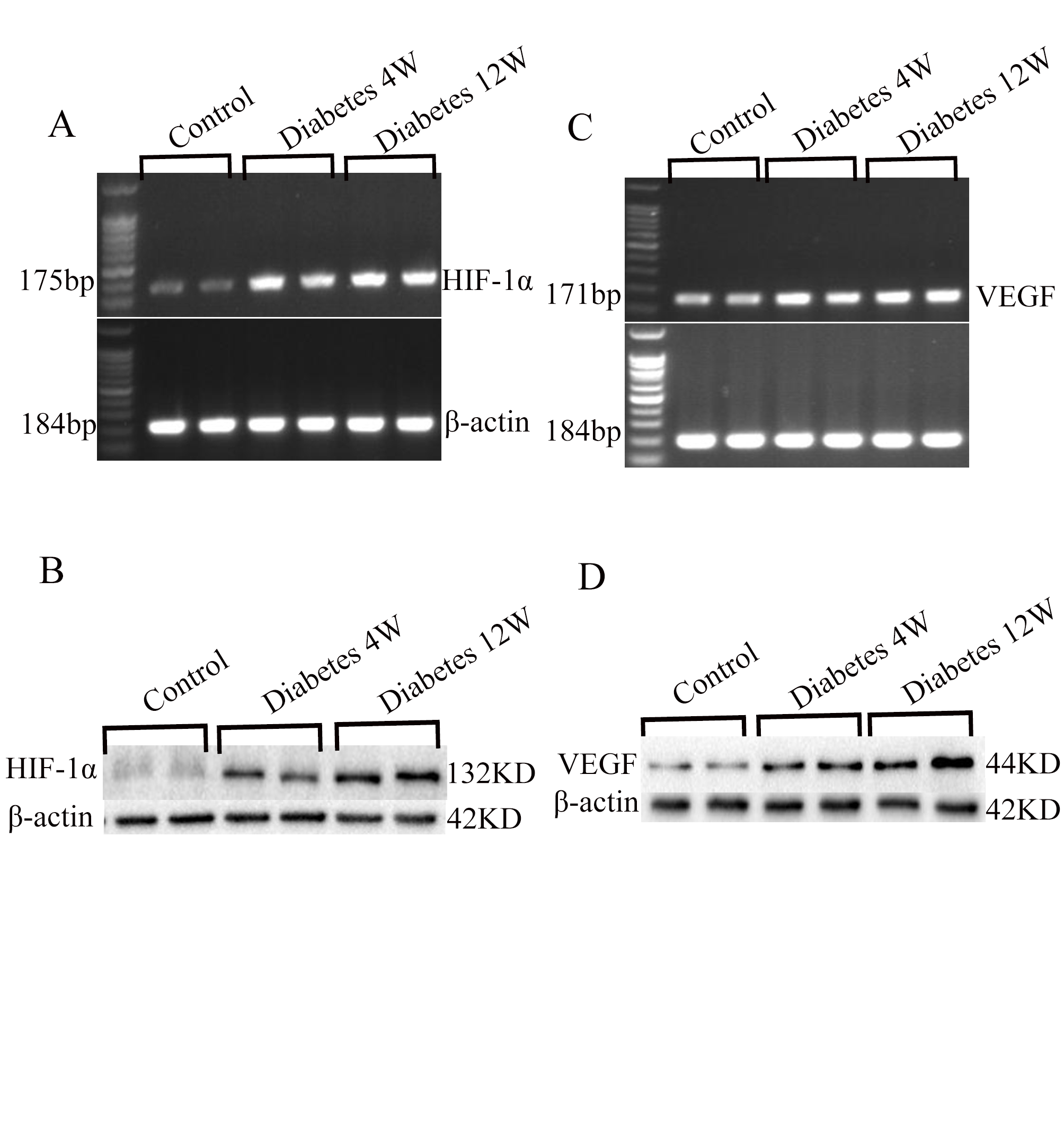

Supplement: Supplementary file 2 — Additional file 2. A. RT-PCR analysis of HIF-1α mRNA expression. B. Western blot analysis of HIF-1α protein expression. C. RT-PCR analysis of VEGF mRNA expression. D. Western blot analysis of VEGF protein expression. [file 12876_2020_1368_MOESM2_ESM.tif]
